# Genetic Characterization of *Microsporum canis* Clinical Isolates in the United States

**DOI:** 10.3390/jof8070676

**Published:** 2022-06-28

**Authors:** Alex Moskaluk, Lauren Darlington, Sally Kuhn, Elisa Behzadi, Roderick B. Gagne, Christopher P. Kozakiewicz, Sue VandeWoude

**Affiliations:** 1 Microbiology, Immunology, and Pathology, Colorado State University, Fort Collins, CO 80523, USA; alex.moskaluk@colostate.edu (A.M.); lauren.darlington@colostate.edu (L.D.); elisa.behzadi@colostate.edu (E.B.); chris.kozakiewicz@colostate.edu (C.P.K.); 2 Behavior Analysis, Simmons University, Boston, MA 02115, USA; kuhnsl@simmons.edu; 3 Pathobiology, Wildlife Futures Program, University of Pennsylvania, Philadelphia, PA 19104, USA; rgagne@vet.upenn.edu

**Keywords:** dermatophytosis, cats, mating-type, *Microsporum canis*, multilocus microsatellite typing

## Abstract

*Microsporum canis* is the primary agent causing dermatophytosis in cats, and also infects humans, dogs, and other species. Assessment of genetic variation among *M. canis* isolates in the United States has not been conducted. Further, *M. canis* mating type and assessment of disease severity associated with genotypic characteristics have not been rigorously evaluated. We therefore isolated *M. canis* from 191 domestic cats across the US and characterized genotypes by evaluation of ITS sequence, MAT locus, and microsatellite loci analysis. The genes SSU1 and SUB3, which are associated with keratin adhesion and digestion, were sequenced from a subset of isolates to evaluate potential genetic associations with virulence. Analysis of microsatellite makers revealed three *M. canis* genetic clusters. Both clinic location and disease severity were significant predictors of microsatellite variants. 100% of the *M. canis* isolates were MAT1-1 mating gene type, indicating that MAT1-2 is very rare or extinct in the US and that asexual reproduction is the dominant form of replication. No genetic variation at SSU1 and SUB3 was observed. These findings pave the way for novel testing modalities for *M. canis* and provide insights about transmission and ecology of this ubiquitous and relatively uncharacterized agent.

## 1. Introduction

*Microsporum canis* is a filamentous fungus that can cause superficial fungal infections in animals and humans [1,2]. It is the primary agent for dermatophytosis cases in domestic cats [1], and for *tinea capitis* in humans in certain parts of Europe [3,4,5]. Since *M. canis* is a zoonotic disease that is a common infection in domestic cats [1,6], it places veterinarians, animal care staff, and owners at risk of infection. Although dermatophytosis is generally self-limiting in immunocompetent individuals, the zoonotic and contagious nature of the infection and its propensity to infect children and medically underserved populations categorize it as an agent of concern [7]. Similar to human infections, feline dermatophytosis presents in a variety of forms [1]. Healthy cats can be presented with areas of circular alopecia and potentially an erythematous margin and scaling [8]. Cats with concurrent infections (e.g., upper respiratory infections), geriatric or feral cats can experience more severe disease with widespread lesions and generalized disease [1]. Cats can be carriers without presenting with lesions, representing a risk for infection to other animals and people [1]. Since *M. canis* is highly contagious and can be difficult to diagnose, outbreaks can frequently occur in high-density populations such as animal shelters [1,9].

Molecular characterization of disease outbreaks can be used to trace pathogen spread and identify contributing factors [10,11,12,13]. Using one microsatellite allele identified by Sharma et al. [14], and seven other loci, Pasquetti et al. [10] identified 22 genotypes from 26 unrelated *M. canis* isolates from 13 non-US countries, including outbreak settings. Transmission between animals and humans was confirmed via identical multilocus genotypes observed in these cases. Understanding the source of *M. canis* infections and pathogen transmission dynamics during outbreaks can inform transmission pathways and mitigate outbreak risk and severity.

In addition to being beneficial in understanding transmission dynamics, genotyping via microsatellite alleles can assess the relationship between clinical presentation and genotype, leading towards better understanding of pathogenesis and providing an opportunity for more informative diagnostic testing [14,15]. Previous studies have demonstrated differences in virulence associated with different *M. canis* genotypes; however, no correlations between genotypes and clinical presentation (or other clinical parameters) have been identified [14,15]. Defining the relationship between clinical presentation and genotype would lead towards elucidation of strain virulence characteristics, and geographic isolate associations could assist in understanding regional risk factors, potentially influencing treatment strategies and prognosis including length of infectivity and disease course.

To our knowledge, genotypic characterization of *M. canis* isolates in the United States has not been conducted. Therefore, we utilized eight microsatellite loci [10,14] to evaluate genetic variants among *M. canis* isolates from cats from seven clinics across the US, characterized the mating gene type and searched for polymorphisms in two candidate *M. canis* virulence genes. We also analyzed demographic, clinic location and clinical presentation data to assess genotypic correlates with these factors and assessed two potential virulence genes for polymorphisms that might relate to pathogenicity.

## 2. Materials and Methods

### 2.1. Sample Acquisition

From May 2019 to June 2021, 258 hair samples were collected from domestic cats with suspected dermatophytosis from animal shelters (California; New Mexico; Boulder Country, Colorado; Larimer County, Colorado; Weld County, Colorado) and veterinary dermatology practices (Massachusetts, New Jersey). Each location consisted of one clinic that was either a shelter or private practice. The criteria for being included in this study were suspicion of dermatophytosis determined by a licensed veterinarian, and no known history of treatment for dermatophytosis. All protocols were approved by Colorado State University (CSU) clinical review board (VCS #2019-223) and biosafety committee (#145399) and were granted an Institutional Animal Care and Use Committee (IACUC) waiver prior to initiation of this study. Samples were collected in shelters upon initial intake examination and were not collected from patients who developed dermatophytosis while housed at the shelter. Veterinarians collected the hair samples using a sterile toothbrush, following the “Mackenzie” brush technique [16,17]. This method was used to ensure sufficient hair was collected from each cat as it is sensitive at acquiring spores from the hair coat [17]. Samples were mailed overnight to CSU, where they were stored at 4 °C until processed.

### 2.2. Clinical Survey

At each participating clinic, veterinarians were provided with an online survey to record clinical and demographic information for each patient sampled. This tool included questions about the individual performing the sampling and analysis, as well as patient ID, date presented for evaluation, age, sex and neuter status, breed, previous and current housing environment, current medications, concurrent medical conditions, diagnostic tests administered, history of anti-fungal treatment, number of lesions present, average size of the lesions, lesion distribution, lesion types, and lesion severity (Table S1). Age was based on dentition for animals with an unknown date of birth. Cats less than one year old were categorized as kittens and over one year old as adults.

### 2.3. Reference Fungal Isolate

*M. canis* isolate CBS 113480 was purchased from Westerdijk Fungal Biodiversity Centre (Utrecht, The Netherlands) and was used as a reference isolate. This isolate was previously used to assemble the published *M. canis* reference genome [18].

### 2.4. Fungal Culture and DNA Extraction

Toothbrush samples were cultured on Dermatophyte Test Medium (DTM) plates (Hardy Diagnostics, Santa Maria, CA, USA) or Sabouraud’s Dextrose Agar (SDA) plates with chloramphenicol and gentamicin (Hardy Diagnostics, Santa Maria, CA, USA) by gently pressing the toothbrush bristles multiple times on each side of the plate. Plates were inverted and incubated at room temperature (20–25 °C) in the dark and were monitored every 24–48 h for colony growth for up to 4 weeks or until fungal growth was detected. Positive dermatophyte culture was determined by colony morphology; if multiple saprophytes were present, samples were re-plated by culturing the toothbrush on a new SDA plate. Plates that were overgrown with saprophytes were re-plated at least two times and observed for dermatophyte growth. All culture negative samples were also replated at least two times.

DNA from fungal cultures were extracted using a protocol developed by Brillowska-Dąbrowska [19]. Fungal cells were transferred via a sterile swab to a tube containing 100 µL of extraction buffer (100 µL of 60 mM sodium bicarbonate, 250 mM potassium chloride, and 50 mM Tris, balanced to pH 9.5). This mixture was incubated at 95 °C for 10 min, followed by addition of 100 µL of 2% bovine serum albumin buffer and then stored at −80 °C until needed.

### 2.5. Dermatophyte Detection and Identification

Qualitative PCR was performed using primers designed specifically for dermatophyte detection that target the ITS-1 gene: DERMF3 (5′-GGTTGCCTCGGCGGGCC) and B-DERMR2 (5′-CGGAATTCTGCAATTCACATTACT) [20]. The following protocol was used for each sample with a reaction volume of 20 µL: 10 µL of 2× KAPA Taq ReadyMix with dye (Roche, Indianapolis, IN, USA), 6 µL UltraPure DNase/Rnase-free distilled water (Invitrogen, Carlsbad, CA, USA), 1 µL of 10 µM forward primer, 1 µL of 10 µM reverse primer and 2 µL of DNA template solution (DNA concentration ranged between 0.1–8.5 ng/µL). The reaction was run in a C1000 Touch thermocycler (Bio-Rad, Hercules, CA, USA) for one cycle at 95 °C for 3 min, 34 cycles at 95 °C for 30 s, 60 °C for 30 s, 72 °C for 1 min, and one cycle at 72 °C for 5 min. Five microliters of PCR product was run on 1.5% agarose gel for 30 min at 80 volts and images were recorded using an ImageQuant LAS 4000 (GE healthcare, Chicago, IL, USA). A 280 bp amplicon was considered positive for dermatophytes. To confirm the identity of the dermatophyte, the PCR products were purified using ExoSAP-IT PCR product cleanup reagent (Applied Biosystems, Carlsbad, CA, USA) and sent for Sanger sequencing (Psomagen, Rockville, MD, USA). The resulting sequences were entered into NIH NCBI’s Basic Local Alignment Search Tool (BLAST) (https://blast.ncbi.nlm.nih.gov/Blast.cgi (accessed on 25 September 2019 to 19 June 2021)).

For determining the mating type gene for the *M. canis* isolates, qualitative PCR using primers that target the alpha-box region (for the MAT1-1 locus) were utilized: Mc_alpha_F (5′-TCTCCTGCTGCCATGGCAACT) and Mc_alpha_R (5′-CAATGGGATTGATGTGGGCA) [21]. PCR and agarose gel conditions were identical to those used for ITS-1 PCR listed above. A 420 bp amplicon was considered positive for the alpha-box gene. Reference strain CBS 113480 was utilized as a positive control as it has been previously identified as belong to the MAT1-1 mating gene type [22].

Qualitative PCR and agarose gel analysis was used to amplify and characterize SSU1 and SUB3 genetic content using the same as detailed above for the ITS-1 PCR, except the annealing temperature was 57 °C for SSU1 and 55 °C for SUB3. Primers for targeting the SSU1 gene were designed in Geneious (v10.0.9, Auckland, New Zealand) and primers for SUB3 were previously designed [23] (Supplementary Materials Table S2). Amplicons between 200 bp to 700 bp were considered positive for SSU1, and a 1149 bp amplicon was considered positive for SUB3. Sequences were compared to reference strain CBS 113480 using Geneious (v10.0.9).

### 2.6. Microsatellite PCR

Eight microsatellite loci (MS) were previously identified and verified in *M. canis* with corresponding primers for these regions [10,14]. To determine the allele sizes for each of the eight microsatellites, Qiagen Multiplex PCR kit (Qiagen, Valencia, CA, USA) was used with 25 µL of Master Mix, 5 µL of 10× primer mix (each primer is at 2 µM concentration), 15 µL of DNase-free water, and 5 µL of DNA (DNA concentration ranged between 0.1–8.5 ng/µL) with a final reaction volume of 50 µL. Multiplex reactions were performed in two separate panels: Panel 1 contained MS 1, 4, 5, and 8, and Panel 2 contained MS 2, 3, 6, and 7 (Table 1). Forward primers were fluorescently labeled on the 5′ end using either 6-FAM, VIC, NED, or PET (Applied Biosystems, Carlsbad, CA, USA) (Table 1). The reaction was run on a C1000 Touch thermal cycler (Bio-Rad, Hercules, CA, USA) for 1 cycle at 95 °C for 15 min, 34 cycles at 94 °C for 30 s, 57 °C for 90 s, 72 °C for 1 min, and 1 cycle at 60 °C for 30 min.

When microsatellites had low quality or unusable results following multiplex PCR, singlet PCR was performed using Phusion High-Fidelity PCR master mix (ThermoFisher Scientific, Waltham, MA, USA) with a 50 µL reaction volume containing 25 µL of 2× Phusion master mix, 15 µL of DNase-free water, 2.5 µL of 10 µM primer F, 2.5 µL of 10 µM primer R, and 5 µL of DNA (DNA concentration ranged between 0.1–8.5 ng/µL). The thermocycler conditions were 98 °C for 30 s, 34 cycles of 98 °C for 10 s, annealing temperature for 30 s, 72 °C for 30 s, and 1 cycle of 72 °C for 10 min. The annealing temperatures used were 60 °C (primers of region 1, 2, 3, 4, 5, and 7), 62 °C (primers of region 8), and 58 °C (primers of region 6). CBS 113480 isolate was run as a positive control [10] and DNase-free water was run as a negative control.

### 2.7. Microsatellite Analysis

PCR product length was assessed using a fragment analyzer (Psomagen, Rockville, MD, USA) and microsatellite allele sizes were classified using Geneious microsatellite plugin (Geneious v10.0.9, plugin v1.4.7) with size standards (GeneScan 500 LIZ, Rockville, MD, USA). To confirm allele calls, each allele was sent for fragment analysis multiple times until at least two high-quality reads produced the same allele size [24]. Loci were considered unusable if low-quality reads were obtained after three replicates.

### 2.8. Statistical Analysis of Genetic Clustering

To evaluate genetic variants among the samples, allele data for each sample were analyzed using the adegenet package (v2.1.3) of R (R version 4.0.4, Vienna, Austria) [25,26]. Principal component analysis (PCA) plots were used to visualize dermatophyte microsatellite variation with respect to clinical and demographic characteristics (i.e., clinic location, sex and neuter status, previous housing, number of lesions, size of lesions, and disease severity). Analysis of variance (ANOVA) was performed on the clinical parameters of location, disease severity, lesion size and number of lesions to determine whether these parameters differed among clinics.

To test associations between clinical parameters and genetic distances among individual isolates, distance-based redundancy analysis (db-RDA) [27] was performed using the function capscale in the vegan package (v2.5.7) [28] of R. Genetic distances were provided as a pairwise matrix of the inverse of the proportion of shared alleles, which were calculated from microsatellite genotypes using adegenet. ANOVA was performed in R on the db-RDA model to determine which of the explanatory variables were significant predictors of genetic distance. To ensure that results were not biased by low sample sizes and thus potentially unrepresentative sampling from some clinics, the ANOVA was repeated using data from California and New Mexico clinics, which had 106 and 26 samples, respectively.

To characterize patterns of dermatophyte genetic structure, allele data were analyzed using two distinct approaches: STRUCTURE (v2.3.4, Stanford University, Stanford, CA, USA) [29] and the snapclust.choose.k function in adegenet (v2.1.3) [25,26]. STRUCTURE implements Bayesian clustering algorithms to estimate the number of distinct clusters (K) that best describes genetic variation among samples and, for each sample, estimates the probability of belonging to each cluster. Ten independent runs were performed for K values one to eight using the admixture model with a burn-in period of 10,000 replications and then 100,000 Markov chain Monte Carlo (MCMC) replications. To determine the optimal K for our dataset, CLUMPAK’s Best K function was utilized [30,31]. This program determines the optimal K value using two methods: one where the uppermost level of structure is found and one where Pr(K = k) is the highest [30,31]. Additionally, the optimal number of clusters was also estimated using snapclust.choose.k, which utilizes goodness-of-fit statistics to identify genetic clusters [25,26]. As before, we tested models for one up to eight genetic clusters.

## 3. Results

### 3.1. Majority of Dermatophytosis Cases Are from Stray Intact Kittens with Multiple Alopecic Lesions

Two hundred and fifty-eight samples were received from seven clinics in five states (165 from California; 53 from New Mexico; eight from Boulder County, CO; 14 from Larimer County, CO; eight from Weld County, CO; eight from Massachusetts; and two from New Jersey); 206 of these were paired with sample survey data (Table 2). Of the received samples, 191 (74%) were culture positive for *M. canis* confirmed by ITS-1 sequencing. 154 of 191 *M. canis* positive samples had accompanying clinical survey data and were included in the study (Table 2). Ninety-seven point four percent of 154 samples (*n* = 150) were collected from shelters and 2.6% (*n* = 4) from individual cases referred by private practitioners. Ninety-six point eight percent of 154 samples (*n* = 149) were from kittens (aged to be younger than one year based on dentition), 89% (*n* = 137) were domestic shorthair (DSH) breed, 89% were intact (*n* = 137) and 89% (*n* = 137) were strays upon presentation. Nearly three quarters (73.6%, *n* = 113) had more than two lesions present and 89% (*n* = 137) had alopecic lesions (Table 3 and Table S3). Most of the lesions were distributed on the head (81%, *n* = 125) and legs including the paws (50%, *n* = 77). The majority of patients (74%, *n* = 114) did not have concurrent medical conditions reported. The most common concurrent medical condition was upper respiratory infection (18.2%, *n* = 28), followed by ectoparasites (3.2%, *n* = 5). In addition, 151 of 154 patients (98.1%) had Wood’s lamp performed before sample collection and 87.4% (132 of 151) were Wood’s lamp positive (Table 3). Based on the clinical presentation, most of the patients were classified with mild (31.8%, *n* = 49) or moderate (25.3%, *n* = 39) disease severity. Clinical survey data were not collected for Weld County, CO samples (*n* = 8), and were not included for New Jersey patients treated with anti-fungal medication before collection of samples (*n* = 2).

### 3.2. Microsatellite Variation among Samples

**Figure 1:**
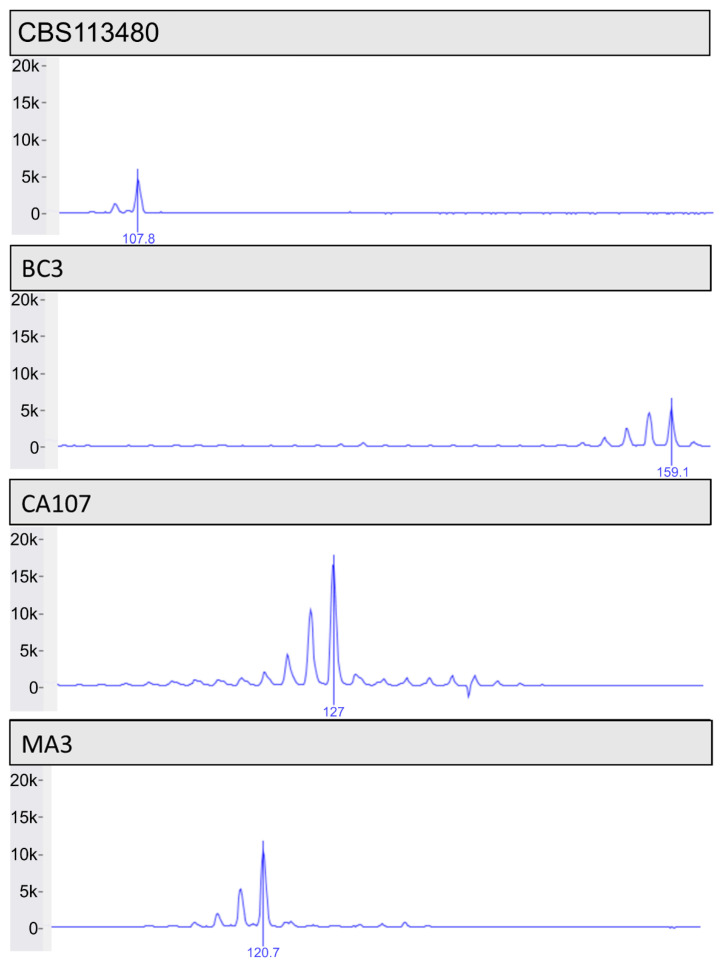
Representative images of microsatellite genotypes. MS4 peaks of unrelated isolates (CBS 113480, BC3, CA107, MA3) showing varying allele sizes at 107 bp, 159 bp, 127 bp, and 121 bp, respectively. CBS = Westerdijk Fungal Biodiversity Centre; BC = Boulder County, CO; CA = California; MA = Massachusetts.

**Figure 2:**
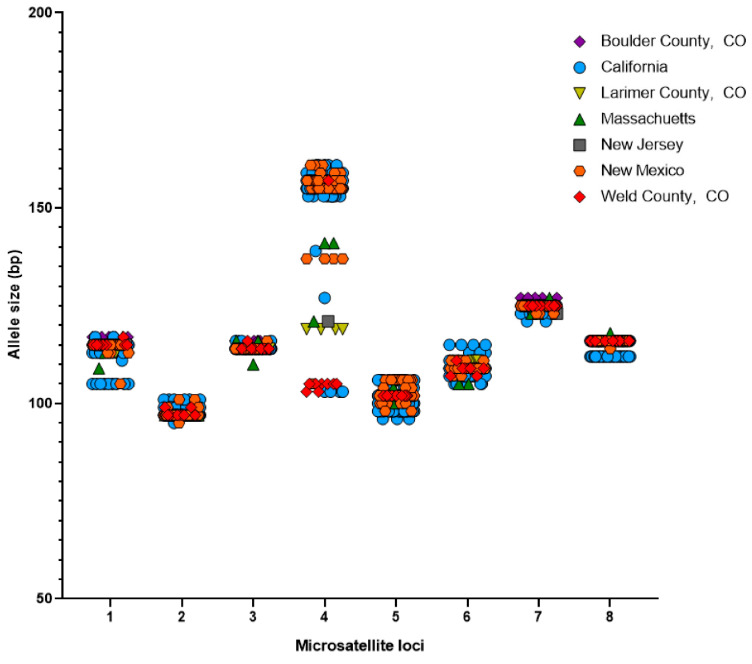
Microsatellite locus allelic diversity varies among *Microsporum canis* isolates. Allele sizes for microsatellite loci (MS) 1–8 for each sample are plotted with Boulder County, CO (*n* = 6); California (*n* = 122); Larimer County, CO (*n* = 4); Massachusetts (*n* = 5); New Jersey (*n* = 1); New Mexico (*n* = 36); Weld County, CO (*n* = 9). The microsatellite loci had the following ranges: 105–117 bp (MS1), 95–101 bp (MS2), 110–116 bp (MS3), 103–161 bp (MS4), 96–106 bp (MS5), 105–115 bp (MS6), 121–127 bp (MS7), and 112–118 bp (MS8).

Of the 191 culture positive samples, 180 were successfully sequenced at six or more microsatellite loci (Figure 1). The number of alleles per MS ranged from 3 (MS3) to 13 (MS4) (Figure 2 and Table S4). Of the 180 genotyped samples, 122 unique multilocus genotypes were observed. There were two genotype combinations observed in nine cats from either California or New Mexico, representing the most frequently observed single genotypes (*n* = 9) (Supplementary Materials Table S4). The genotype combination of reference strain CBS 113480 was not observed amongst the clinical samples (Table S4). MS4 had the greatest variability with most frequently observed allele occurring in 43% of the samples, while MS3 showed the lowest variation, with the majority of samples (79%) having a 114 bp allele at this locus (Figure 2 and Table 4). The other MS ranged in size from 105 bp to 117 bp (MS1), 95 bp to 101 bp (MS2), 96 bp to 106 bp (MS5), 105 bp to 115 bp (MS6), 121 bp to 127 bp (MS7), and 112 bp to 118 bp (MS8) (Table 4). Across all MS, the percent of missing alleles per locus ranged from 0% (MS2, 5) to 21% (MS6) (Table 4).

### 3.3. All M. canis Isolates Expressed the MAT1-1 Mating Type Locus

For the 180 *M. canis* isolates that were utilized for microsatellite genotyping, qualitative PCR for the mating locus alpha-box gene was performed. An amplification product was observed at 420 bp for each isolate, indicating that all isolates were of the negative mating gene type (MAT1-1) (Table S4).

### 3.4. Homologous Sequences for SSU1 and SUB3 between Isolates

Of the 180 *M. canis* isolates utilized for the microsatellite PCRs, the SSU1 genes were characterized for 46 isolates from varied sites including Boulder (*n* = 3); New Mexico (*n* = 7); California (*n* = 24); Larimer County, CO (*n* = 3); Weld County, CO (*n* = 6); Massachusetts (*n* = 2); and New Jersey (*n* = 1) (NCBI GenBank accession numbers ON799017-62). Six isolates from California were evaluated for SUB3 gene sequence (NCBI GenBank accession numbers ON755064–68). The sequences for SSU1 and SUB3 for all isolates were 100% identical to reference strain CBS 113480 [18,23].

### 3.5. Clinic Location and Disease Severity Were Associated with Microsatellite Genotype

**Figure 3:**
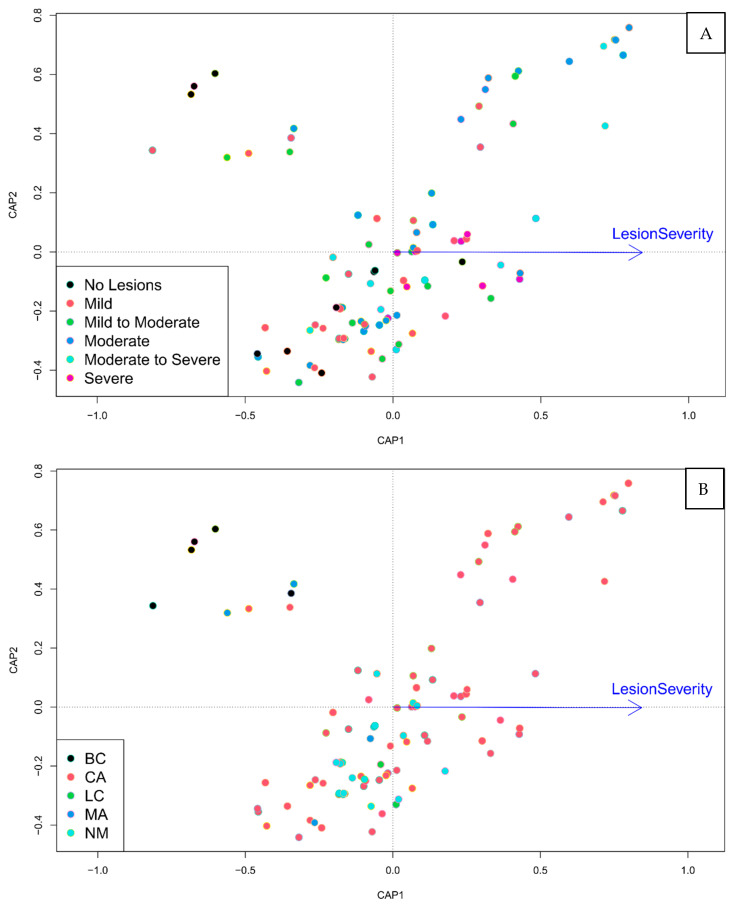
Disease severity and location associated with microsatellite genotype. Distance-based redundancy analysis showed significant association between disease severity and microsatellite variation. (**A**)—samples colored based on clinic location. (**B**)—samples colored by disease severity. Locations included in study are California (CA); New Mexico (NM); Boulder County, CO (BC); Larimer County, CO (LC); and Massachusetts (MA). Position on the plot for each sample depicts genetic differentiation. Arrow = disease severity increases direction of arrow.

db-RDA modeling using ANOVA indicated significance among clinical parameters (*p*-value = 0.001, Figure 3). Both clinic location (*p*-value = 0.001) and disease severity (*p*-value = 0.004) were found to be significant predictors of genetic distance (Figure 3 and Table S5). To test the correlation between disease severity and location, we performed an ANOVA and found that disease severity differed among locations (*p*-value = 0.0049) with number of lesions and size of lesions not varying significantly among locations (*p*-value = 0.34 and *p*-value = 0.57, respectively). However, when db-RDA analysis included only clinics with large sample sizes (California and New Mexico), we found that the association between disease severity and genetic distance was still significant (*p*-value = 0.012 and *p*-value 0.011, respectively, Table S6).

**Figure 4:**
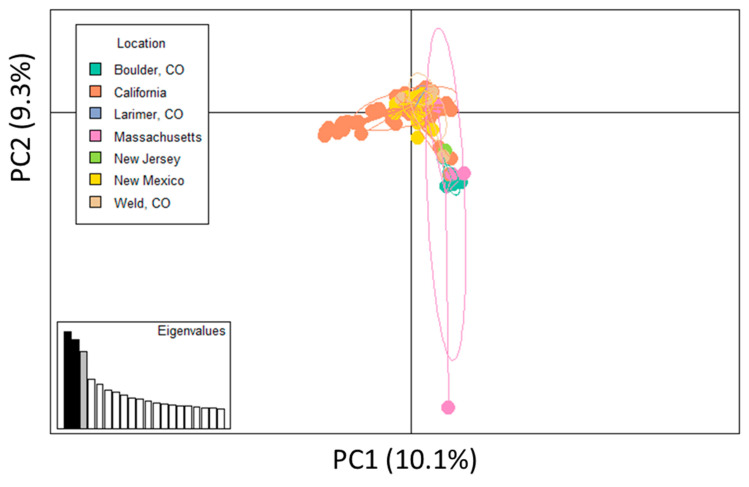
Evidence of separation based on clinic location for Boulder, Massachusetts, and part of California. Principal component analysis (PCA) plot for microsatellite regions, with samples colored according to clinic. One sample from Massachusetts was an outlier. The portion of California samples that appear distinct were collected in the same month. Locations included in study are California; New Mexico; Boulder County, CO; Larimer County, CO; Weld County, CO; New Jersey; and Massachusetts.

PCA plots were used to summarize *M. canis* genetic variants with respect to six parameters, including: (1) geographic location of clinic, (2) sex/neuter status, (3) clinical disease severity, (4) number of lesions present, (5) lesion(s) size, and (6) previous housing environment. Three distinct genetic groups were identified in relation to clinic of origin. Samples from the Boulder County, CO and Massachusetts appeared genetically distinct from other sites (Figure 4). One sample from Massachusetts was distinct from all other samples. This sample was coincidentally the only Persian cat analyzed in this study (Figure 4, Table S3). The third genetic group contained samples from California, where the majority of the samples clustering had been collected during the same month.

### 3.6. Genetic Clustering of M. canis Isolates

**Figure 5:**
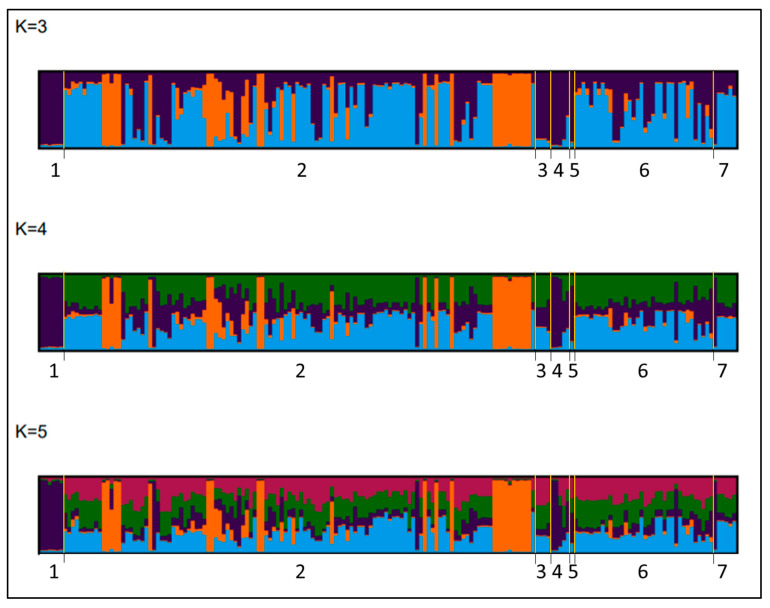
Samples from Boulder County, CO and a portion of California clustered apart from other sampled locations. Structure plots with K values 3–5. The amount of vertical color for each sample corresponds with the percentage of belonging to that color cluster. Admixture increased for plots using K = 4 and K = 5. Locations included in study were Boulder County, CO (1); California (2); Larimer County, CO (3); Massachusetts (4); New Jersey (5); New Mexico (6); and Weld County, CO (7).

STRUCTURE analysis indicated the greatest support for three distinct genetic clusters (K = 3) according to both the ΔK and prob(K) metrics (Figure S1). However, snapclust.choose.k indicated five genetic clusters (K = 5; Figure S1). To resolve this discrepancy, we scrutinized plots showing genetic cluster membership probabilities of individual *M. canis* isolates for K = 3–5 (Figure 5). We found that neither the K = 4 nor K = 5 models revealed any additional genetic structure among samples that was not evident with K = 3, suggesting that K = 3 was the most biologically informative model (Figure 5). Because of this, and because K = 3 was most consistent with the PCA results, we concluded that there were most likely three genetic clusters among our *M. canis* isolates. The three *M. canis* genetic clusters appeared to be partly associated with different clinics (Figure 5). Samples from the Boulder County, CO; Larimer County, CO; Massachusetts; and New Jersey clinics clustered in one group (dark blue/purple, Figure 5). California samples comprised two genetic clusters (orange, light blue, Figure 5). The New Mexico and Weld County, CO clinics comprised samples that assigned to both the California and Boulder genetic clusters (Figure 5). Genetic clusters were not related to sex/neuter status, previous housing environment, number of lesions, size of lesions, and disease severity (Figures S2–S6).

## 4. Discussion

Few studies have assessed genotypic or regional factors that influence dermatophyte clinical presentation [14,32,33]. These studies have utilized microsatellite DNA polymorphisms as this is an established method for genotypic comparisons among large sample sets and have been identified in dermatophytes [10,14]. With two microsatellite loci, 101 *M. canis* isolates were classified as three distinct populations [14]. One of these populations was found to be over-represented in human samples, implying that this group has a greater ability to infect humans due to higher virulence in humans [14]. Clinical presentations were classified based on anatomical location for human samples (e.g., *tinea capitis*, *tinea corporis*, etc.) whereas cases in animals were characterized as ringworm [14]; however, no associations between genotype and clinical presentation were identified [14].

Da Costa et al. [15] utilized two microsatellite loci identified by Sharma et al. [14] to analyze 102 *M. canis* isolates from Brazil isolated from humans, cats, and dogs. Fourteen distinct multilocus genotypes and six distinct populations were identified [15]. Genotypes were compared to age, sex, breed, symptomatology, geographical location, and living conditions, and broadly characterized symptoms (asymptomatic, focal ringworm, multifocal ringworm, ringworm, and pseudomycetoma) [15]. No correlations were noted between genotypes at two microsatellite loci and these parameters [15]. Notably, these previous attempts to associate genotypes with clinical parameters had limited statistical power owing to the use of a very small number of loci. Further investigation using a greater number of loci is necessary to quantify genetic variants with sufficient precision to identify associations with clinical parameters.

Expanding upon previous microsatellite studies, our results demonstrated that disease severity is associated with genetic distance of *M. canis* isolates, suggesting that *M. canis* genetics play a significant role in clinical presentation. Understanding this difference in virulence between genotypes could be beneficial in implementing treatment plans as strains that cause more severe disease might require more intensive therapy, particularly if the patient has co-morbidities. Previous studies of dermatophytosis have demonstrated more severe inflammatory infections generally resolve faster, suggesting that the host immune system is effective at clearing the infection [34]. Knowing that certain *M. canis* genotypes can cause more severe disease can help clinicians better predict when infections will resolve. Although we identified genetic differentiation with respect to disease severity, it is unlikely that the MS analyzed are associated with fungal virulence genes as these loci are not associated with specific gene products. However, our results suggest that variation in *M. canis* virulence may be at least partly genetically determined. Further studies implementing more extensive genome-wide sequencing approaches are warranted to identify the genetic basis of *M. canis* virulence.

Genetic clustering was observed with respect to clinic location, suggesting that location or clinic-specific genotypes of *M. canis* may occur. This finding may represent *M. canis* spread within a specific site or setting. As the sample size for five of seven locations was less than 10 individuals per clinic, it is unlikely that samples from these clinics are truly representative of the genetic variants present in the surrounding regions. These regions that had clinic locations align with the clusters (Boulder County, Massachusetts, and California) have different climates, and represent private practices (Massachusetts) and shelters (Boulder, California), further suggesting that the observed clustering has clinic-specific factors involved. Samples from the Massachusetts clinic were collected over months from different private owners, suggesting that the genotype was more region-specific, since these cats with ringworm were unlikely to have been exposed to one another.

We noted one highly divergent *M. canis* genotype isolated from the only sample from a Persian cat included in this study. Persian cats have been reported to have a higher prevalence of dermatophytosis [35,36], although it has been suggested that it is due to sampling bias [17]. Long-haired cats (including Persians) have also been shown to have higher rates of subclinical dermatophytosis due to their longer coats [37]. Further sample collection from Persian and other long-haired cats could elucidate if certain *M. canis* genotypes preferentially infect these breeds and if clinical disease differs in these infections.

The reference strain CBS 113480 demonstrated a different genotype that was not observed in our data set, with two loci containing novel alleles. While our samples were from domestic cats in the Unites States, this reference strain was originally isolated from a human in Germany, suggesting that different *M. canis* strains could be present in the United States compared to in Europe, and that strains circulating on cats could be different from ones infecting humans. The allele values for the eight microsatellite loci for the reference strain have been previously published and are slightly different from our genotype results, likely due to differences in equipment and software used for genotyping inherent in microsatellite analysis [38,39].

Given that dermatophytes can utilize both sexual and asexual modes of reproduction [21,33,40,41], identifying the mating types for different isolates can help understand genetic mixing in the population. Dermatophytes have two mating types, positive and negative, which correspond to the mating type genes high mobility group and alpha-box, respectively [21]. Understanding dermatophyte mating types can reveal if populations have shifted toward one form of reproduction and the possible effect this can have on the genotypes present, which is particularly important for tracking virulence genes.

For our study, all samples that were utilized for microsatellite analysis were determined to possess the MAT1-1 mating locus, suggesting that this mating locus is dominant in the United States, making *M. canis* more reliant on asexual reproduction. It has been previously suggested that the MAT1-2 mating locus for *M. canis* has become extinct [21] with the only known MAT1-2 *M. canis* isolates originating from Japan [42]. The extinction of one mating type and loss of sexual reproduction has been documented for various other dermatophyte species, including *Trichophyton rubrum* morphotype “megninii”, *Trichophyton tonsurans*, *Trichophyton interdigitale*, *Trichophyton schonleinii*, *Trichophyton concentricum*, *Microsporum audouinii*, and *Microsporum ferrugineum*, all of which are anthropophilic [21]. It has been suggested that this shift in mating type distribution reflects the fungi becoming more adapted to a particular host species, leading to a loss of interaction between the two mating types [21]. This shift could suggest that *M. canis* is becoming more adapted to cats as they are the primary host, indicating that infections could become more chronic and less inflammatory, similarly to infections caused by anthropophiles that rely on asexual reproduction [1,21,43].

Two potential virulence genes for *M. canis* include sulfite efflux pump (SSU1) and subtilisin 3 (SUB3) as these genes are critical for the beginning stages of infection [44,45,46,47]. Both genes were 100% identical to the reference isolate CBS 113840 for the samples analyzed, suggesting that these genes are conserved between isolates. Due to the importance of these genes for establishing infections, mutations in these genes would likely be deleterious, reducing the fungus’ chances of survival. Exploring the level of expression of these genes could elucidate the potential differences of clinical presentation for various isolates.

In addition to genetics, another interesting component of our work investigated the demographics of the study populations. Kittens are known to have higher rates of *M. canis* clinical infection [1,17], a finding represented in our study. Because our sample set was strongly biased to clinical samples from shelter animals, this undoubtedly affected our findings, though higher rates of infection in kittens compared to adult cats generally is thought to be related to maturation of the immune system [48]. While the immune system is considered competent by 6–12 weeks of age in cats, the cellular processes critical for eliminating dermatophytes (the maturation of phagocytic and antigen-presenting cells) have not been studied [48]. Mature versions of these phagocytic and antigen-presenting cells are responsible for removing dermatophytes from the host through extra/intracellular lysis of the fungi and cytokine production [49]. However, immature versions of these cells could have a delayed or less potent response during the beginning stages of infection, preventing kittens from quickly eliminating infection before clinical signs develop. Further investigation into immature immune systems of cats could help elucidate key differences that occur in neonatal and adolescent cats, particularly during fungal infections.

The majority of patients with clinical data reported positive fluorescence under a Wood’s lamp, suggesting that Wood’s lamp is an effective screening tool for *M. canis* infections in cats. The reported percentage of *M. canis* strains that fluoresce following Wood’s lamp examination ranges from 45.5% [2] to 100% [50]. Reports from the 1950s associated fluorescence of *M. canis* with production of the compound pteridine [51,52]. It is unclear if different isolates have varying ability to produce pteridine or other fluorescent metabolites, or if this metabolite is only produced during certain stages of infection. The metabolic pathway that produces pteridine and the precursor metabolites have not been investigated. Future studies of pteridine and associated metabolic pathways could help explain the function of this product and whether it represents a potential target for a metabolic-based diagnostic assay.

## 5. Conclusions

Our work represents the first comprehensive assessment of *M. canis* genetic and clinical associations conducted in the US. Our analysis showing that a small handful of microsatellite loci are capable of distinguishing geographic and virulence characteristics provides great optimism that more rigorous genomic comparisons will reveal markers that can be exploited to diagnostic and therapeutic advantage. Disease severity was demonstrated to be a significant predictor of genetic differentiation among *M. canis* isolates. Our finding of MAT1-1 locus homogeneity suggests evolution of domestic cat-associated *M canis* towards a largely asexual reproduction cycle, with significant ecological and biological implications, and again offering potential therapeutic targets for this widespread zoonotic agent. Other genes essential for adhesion and keratin metabolism (SUB3, SSU1) were evaluated in a subset of the cohort and were found to be genetically identical across clinic locations.

In summary, our findings suggest *M. canis* isolates are genetically distinct, and specific genotypes may have a range of phenotypic presentations. Follow on studies exploring more comprehensive sequencing methods, such as whole genome sequencing, provide an exciting avenue for in-depth genetic analyses that could reveal differences in clinical presentation, unknown functions inherent in superficial fungal metabolism, and may suggest novel and innovative therapeutic modalities.

## Figures and Tables

**Table 1:** Primer sequences with corresponding fluorescent dyes (6-FAM, VIC, NED, and PET) used for the microsatellite loci.

Panel	Microsatellite Locus	Orientation	Primer Sequence 5′ to 3′	Reference
1	1	Forward	[VIC]GAAGGAGGTATATATGGGTGTG	[10]
		Reverse	GATAAGGTGTTTGGCACTGA	
	4	Forward	[6-FAM]CAGCATCTAAATAACTGGCCTA	[10]
		Reverse	TTTTCTTTCTACTTCCCGTTG	
	5	Forward	[NED]GGTTTACACGCAGCATGA	[10]
		Reverse	CGTGGCTGAAGAAGTCTACC	
	8	Forward	[PET]GATCGGAGCATGCCATACAG	[14]
		Reverse	TCTTCCCACCCTTCTCAATG	
2	2	Forward	[NED]GGGAACAATCTGCCTTAAAC	[10]
		Reverse	CACAGAGATATGCCGTATGC	
	3	Forward	[PET]AGGTGTTTGGCACTGAGC	[10]
		Reverse	CGAAGAGAAGGAGGTATATATGG	
	6	Forward	[6-FAM]CGTCTGGGACTTGGTAGTAA	[10]
		Reverse	TCGGAGGATCTTTAAACTGT	
	7	Forward	[VIC]GCCAAAGAGCTTGCTGAG	[10]
		Reverse	CGTTAGCATGCATCTCTCTATAC	

**Table 2:** One hundred forty-seven samples were included in microsatellite analysis associated with reported clinical attributes.

Sample Categories	CA	NM	BC	LC	WC	MA	NJ	Total
Samples received	165	53	8	14	8	8	2	**258**
Culture positive samples	132	42	6	4	6	5	1	**191**
Samples with clinical data	139	36	8	14	0	7	2 *	**206**
Culture + clinical data	109	31	6	4	0	4	1 *	**155**
Microsatellite samples	122	36	6	4	6	5	1	**180**
Microsatellite + clinical data	106	26	6	4	0	4	1 *	**147**

* clinical data from New Jersey samples were excluded from downstream analysis due to the patients being treated from dermatophytosis prior to sample collection. Culture + clinical data samples are samples that were culture positive for *Microsporum canis* and had corresponding clinical data for the patient. Microsatellite + clinical data samples are samples that were successfully sequenced for the microsatellite regions and had corresponding clinical data for the patient. CA = California; NM = New Mexico; BC = Boulder County, CO; LC = Larimer County, CO; WC = Weld County, CO; MA = Massachusetts; NJ = New Jersey.

**Table 3:** Summary of clinical presentation data for *Microsporum canis* positive cats.

Clinical Parameters	Total (*n* = 154)
Sample from shelter or private practice	Shelter (150, 97.4%)
	Private practice (4, 2.6%)
Age	Kitten (149, 96.8%)
Sex	Female (74, 48.1%)
Neuter status	Intact (138, 89.6%)
Breed	DSH (137, 89%)
	DMH (11, 7.1%)
	DLH (4, 2.6%)
	Persian (1, 0.6%)
	Exotic Shorthair (1, 0.6%)
Previous housing environment	
Stray	1 (138, 89.6%)
Single cat—owned household	2 (0, 0%)
Multi cat—owned household	3 (3, 1.9%)
Previously owned—other animals unknown	4 (2, 1.3%)
Transferred from shelter	5 (11, 7.1%)
Current housing environment	
Shelter/rescue—single cat kennel	1 (40, 26%)
Shelter/rescue—multiple cat kennel	2 (109, 70.8%)
Client owned—single cat household	3 (1, 0.6%)
Client owned—multiple cay household	4 (4, 2.6%)
Current medications *	
Yes—topical parasite preventatives or dewormer	1 (32, 20.8%)
Yes—oral parasite preventatives or dewormer	2 (57, 37%)
Yes—other	3 (2, 1.3%)
No	4 (83, 53.9%)
Concurrent medical conditions *	URI (28, 18.2%)
	Ectoparasites (5, 3.2%)
	Superficial pyoderma (1, 0.6%)
	IBD (1, 0.6%)
	Diarrhea (1, 0.6%)
	Otitis media (1, 0.6%)
	Degloved chin (1, 0.6%)
	Underweight (1, 0.6%)
	None (114, 74%)
Diagnostics performed *	
Wood’s lamp	1 (151, 98.1%)
Fungal culture	2 (10, 6.5%)
IDEXX PCR	3 (4, 2.6%)
Hair observed under microscope	4 (2, 1.3%)
Other	5 (3, 1.9%)
None	6 (1, 0.6%)
If diagnostics have been performed, what were the results?	Wood’s lamp positive (132, 87.4%)
Number of lesions	
1	1 (29, 18.8%)
2–5	2 (67, 43.5%)
5+	3 (46, 29.9%)
None—no lesions present	4 (12, 7.8%)
Size of lesions	
Less than 0.5 inch (smaller than a dime)	1 (62, 40.2%)
Between 0.5 inch and 1 inch (size of a penny)	2 (59, 38.3%)
Greater than 1 inch (larger than a penny)	3 (21, 13.6%)
N/A—no lesions present	4 (12, 7.8%)
Lesion distribution *	
Head	1 (124, 80.5%)
Neck	2 (46, 30%)
Abdomen	3 (46, 30%)
Back	4 (31, 20.1%)
Tail including base of tail	5 (28, 18.2%)
Legs including paws	6 (77, 50%)
N/A—no lesions present	7 (12, 7.8%)
Lesion types *	
Alopecia	1 (137, 89%)
Scales/crusts	2 (95, 61.7%)
Reddened/erythematous	3 (9, 5.8%)
Papules/pustules	4 (0, 0%)
Miliary dermatitis	5 (2, 1.3%)
N/A—no lesions present	6 (12, 7.8%)
Disease severity	
Mild—lesions are small and confined to focal area	1 (49, 31.8%)
Mild to moderate	2 (20, 13%)
Moderate—lesions are found in multiple areas, but is not widespread	3 (39, 25.3%)
Moderate to severe	4 (14, 9.1%)
Severe—lesions are large and found all over the body	5 (20, 13%)
N/A—no lesions present	6 (12, 7.8%)

* = Percentages are greater than 100 as patients can have more than one answer. DSH = domestic shorthair, DMH = domestic medium hair, DLH = domestic long hair, N/A = not applicable, URI = upper respiratory infection, IBD = inflammatory bowel disease.

**Table 4:** Eight microsatellite loci demonstrate different degrees of polymorphism across samples tested.

Microsatellite Locus	Allele Size Range	Most Common Allele (% of Alleles)	% of Missing Genotypes
1	105–117 bp	115 bp (72%)	7.2%
2	95–101 bp	97 bp (78%)	0%
3	110–116 bp	114 bp (79%)	13%
4	103–161 bp	155 bp (43%)	1.7%
5	96–106 bp	102 bp (47%)	0%
6	105–115 bp	109 bp (48%)	21%
7	121–127 bp	125 bp (81%)	2.2%
8	112–118 bp	116 bp (79%)	2.2%

## Data Availability

All data are contained within the article and Supplementary Materials.

## Supplementary Materials

The following supporting information can be downloaded at: https://www.mdpi.com/article/10.3390/jof8070676/s1, Figure S1: Clinic location and disease severity were significantly associated with genetic distance; Figure S2: Genetic clustering is not observed among categories of disease severity; Figure S3: Genetic clustering is not observed among categories of number of lesions; Figure S4: Genetic clustering is not observed among categories of number of lesions; Figure S5: Genetic clustering is not observed among categories of previous housing environment; Figure S6: Genetic clustering is not observed among categories of sex and neuter status; Table S1: Survey provided to shelters and clinics for suspected dermatophytosis patients; Table S2: Primers utilized for characterizing SUB3 and SSU1 genes; Table S3: Clinical presentation data for *Microsporum canis*-positive cats from CA, NM, CO (Boulder, Larimer), and MA; Table S4: Allele sizes for microsatellite regions one to eight (MS1, MS2, MS3, MS4, MS5, MS6, MS7, MS8) for *Microsporum canis* samples; Table S5: Clinic location and disease severity were significantly associated with genetic distance; Table S6: Clinic location and disease severity were significantly associated with genetic distance.
