# Supplementary material for: Genetic Characterization of Microsporum canis Clinical Isolates in the United States
Source: J Fungi (Basel). 2022 Jun 28;8(7):676. doi: 10.3390/jof8070676 (PMC9321804; doi:10.3390/jof8070676)
Supplement: Supplementary file 1 [file jof-08-00676-s001.zip › Supplementary Figures Moskaluk 2022.pdf]

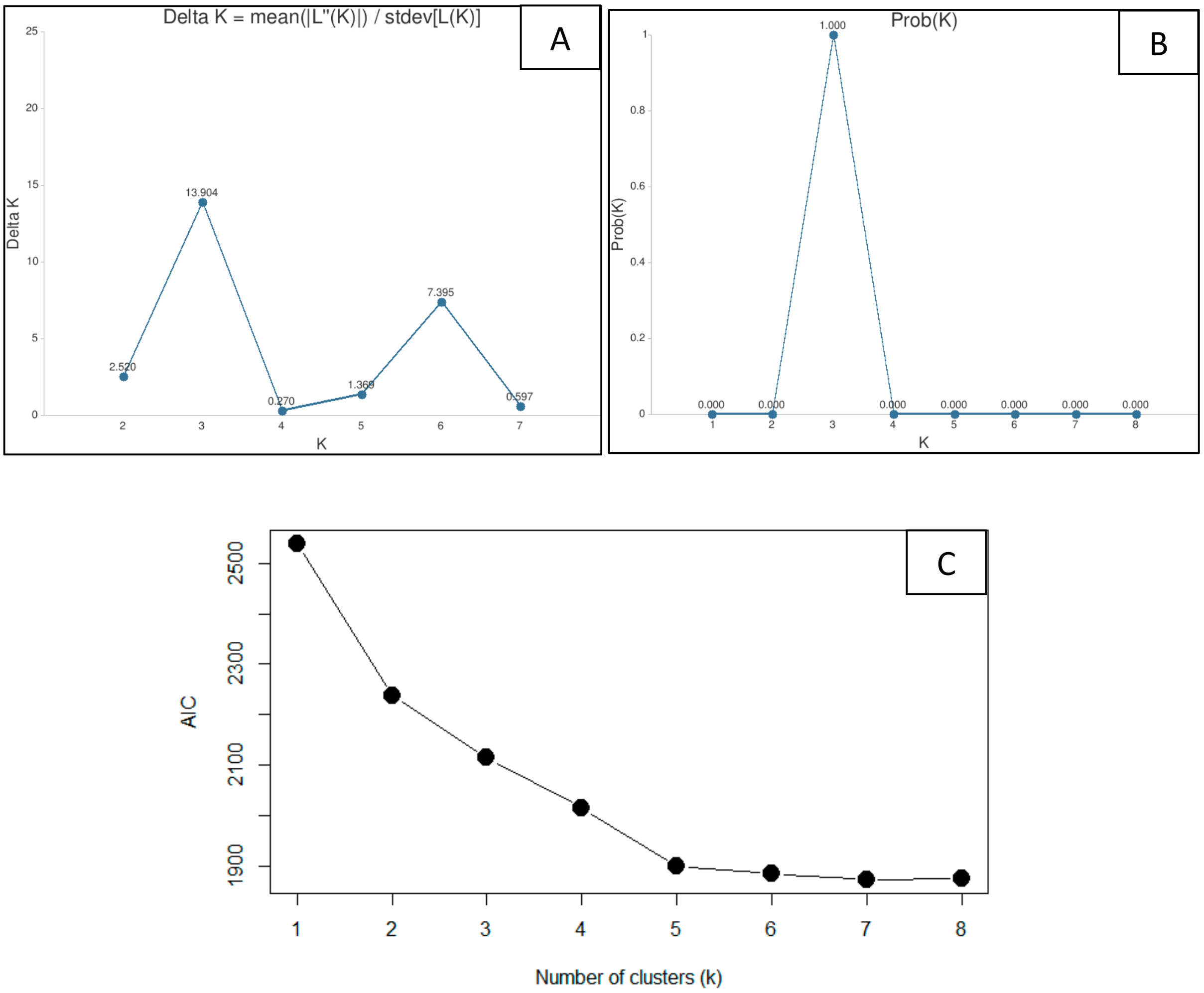

Figure S1. Optimal clustering of study population ranged between 3 and 5. (A & B) Optimal K output graph generated by CLUMPAK from STRUCTURE results according to delta K and Prob(K) support. Both approaches indicate that K = 3 is the most supported model for explaining genetic structure among *Microsporum canis* isolates. (A) The optimal K was 3 using delta K = mean(lL"(K)l)/stdev[L(K)]. (B) The K for which Pr(K=k) is the highest was 3. (C) Optimal k output graph generated by snapclust.choose.k program. AIC support increased with successive value of k up until 5, after which AIC support did not increase, suggesting that k = 5 is the most supported model.

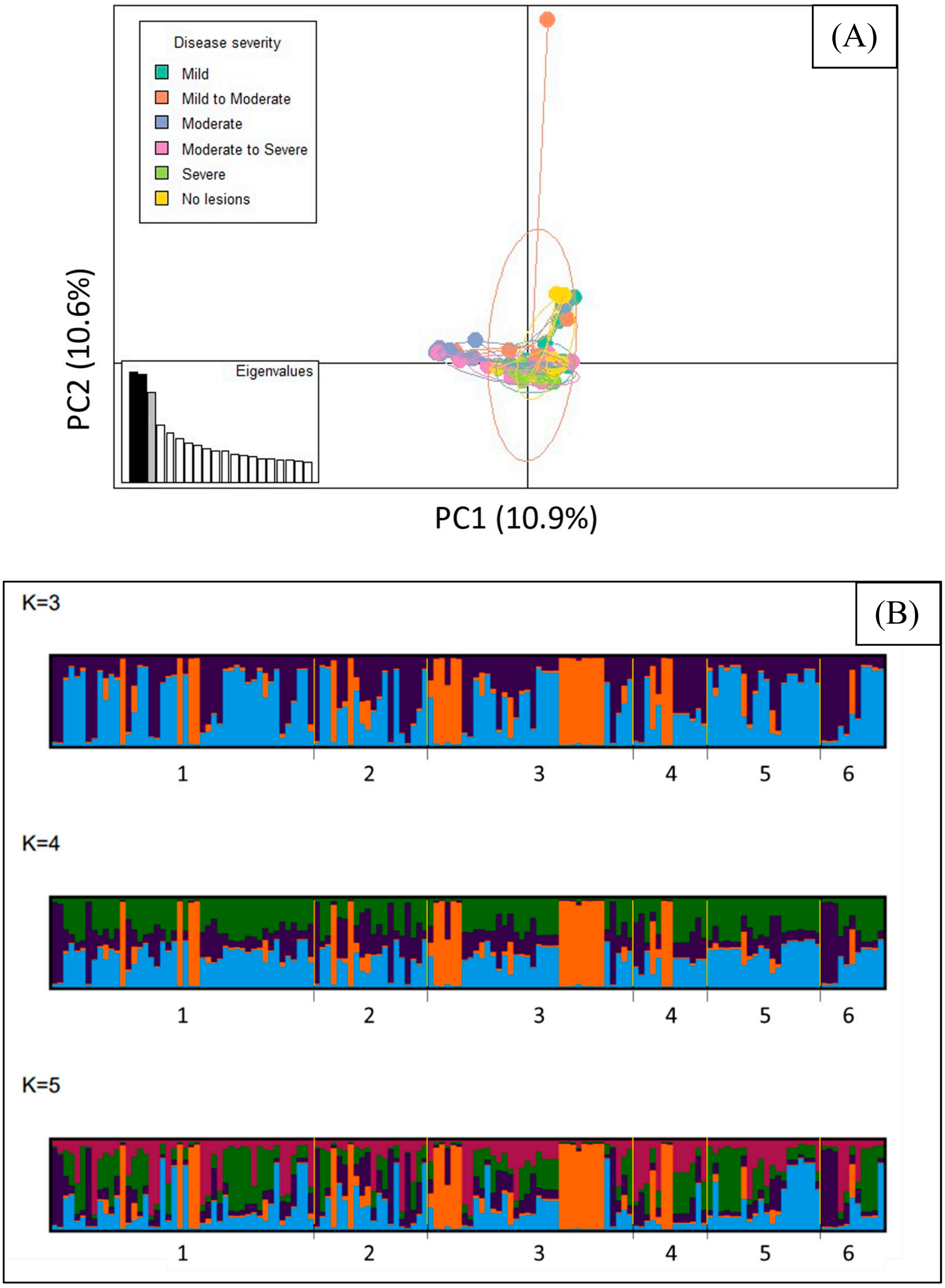

Figure S2. Genetic clustering is not observed among categories of disease severity. (A) PCA plot for microsatellite regions using disease severity collected from the clinical survey. The outlier point for group mild to moderate was a sample from MA. (B) Structure plots with K values 3-5. The amount of vertical color for each sample corresponds with the percentage of belonging to that color cluster. Admixture increased for plots using K=4 and K=5. Disease severity groups included in study were mild (1), mild to moderate (2), moderate (3), moderate to severe (4), severe (5), and no lesions (6). Locations included in study were California; New Mexico; Boulder County, CO; Larimer County, CO; and Massachusetts.

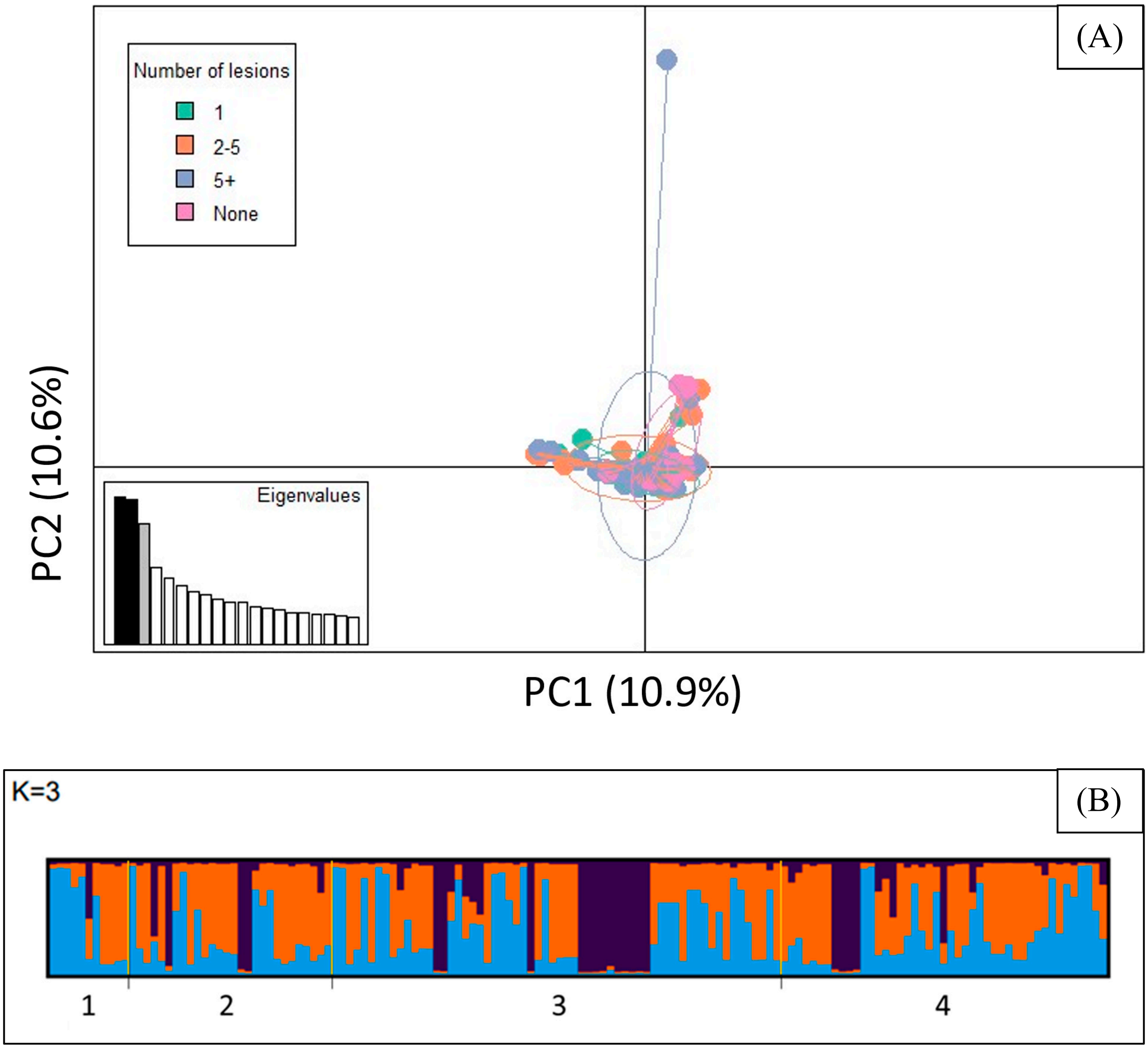

Figure S3. Genetic clustering is not observed among categories of number of lesions. (A) PCA plot for microsatellite regions using the number of lesions (if present) collected from the clinical survey. The outlier point for 5+ lesions group was a sample from MA. (B) STRUCTURE plot (k=3) grouping samples based on number of lesions where 1 = none, 2 = 1 lesion, 3 = 2-5 lesions, and 4 = 5+ lesions. Locations included in study were California; New Mexico; Boulder County, CO; Larimer County, CO; and Massachusetts.

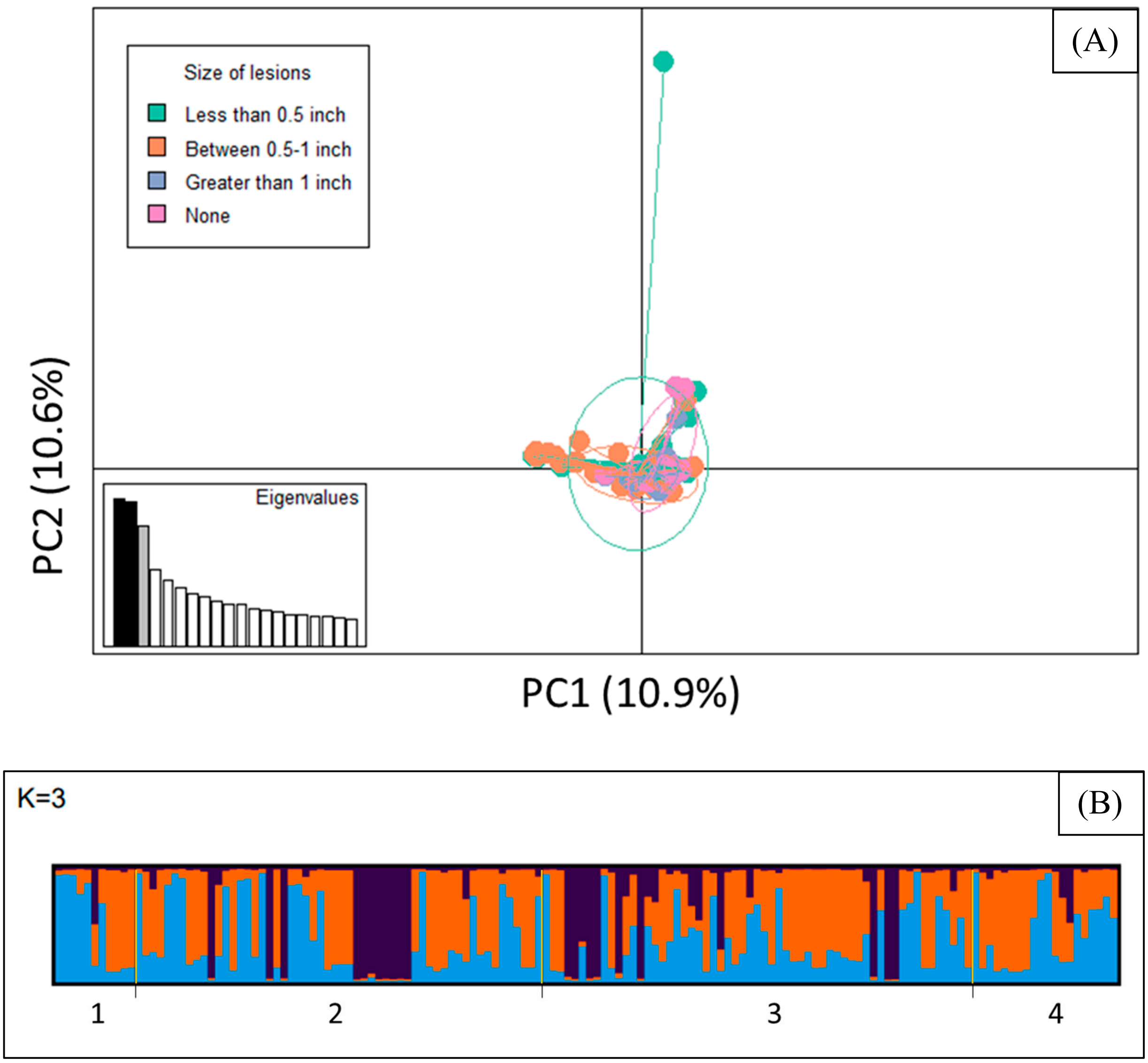

Figure S4. Genetic clustering is not observed among categories of size of lesions. (A) PCA plot for microsatellite regions using the size of the lesions (if present) collected from the clinical survey. The outlier point for less than 0.5 inch group was a sample from MA. (B) STRUCTURE plot (k=3) grouping samples based on size of lesions where 1 = none, 2 = less than 0.5 inch, 3 = between 0.5 inch and 1 inch, and 4 = greater than 1 inch. Locations included in study were California; New Mexico; Boulder County, CO; Larimer County, CO; and Massachusetts.

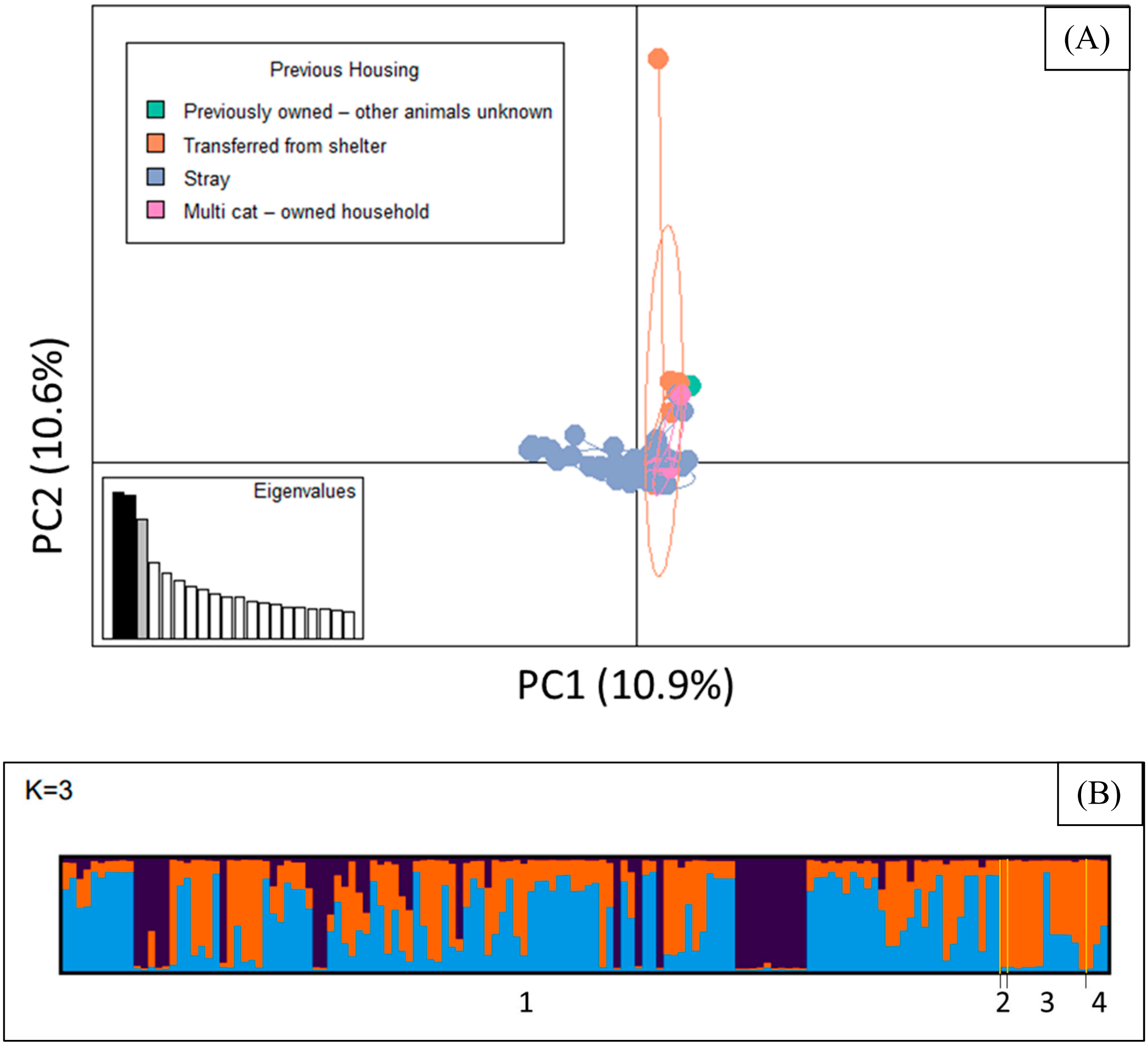

Figure S5. Genetic clustering is not observed among categories of previous housing environment. (A) PCA plot for microsatellite regions using the previous housing environment collected from the clinical survey. The outlier point for transferred from shelter group was a sample from MA. (B) STRUCTURE plot (k=3) grouping samples based on previous housing environment where 1 = stray, 2 = previously owned – other animals unknown, 3 = transferred from shelter, and 4 = multi cat – owned household. Locations included in study were California; New Mexico; Boulder County, CO; Larimer County, CO; and Massachusetts.

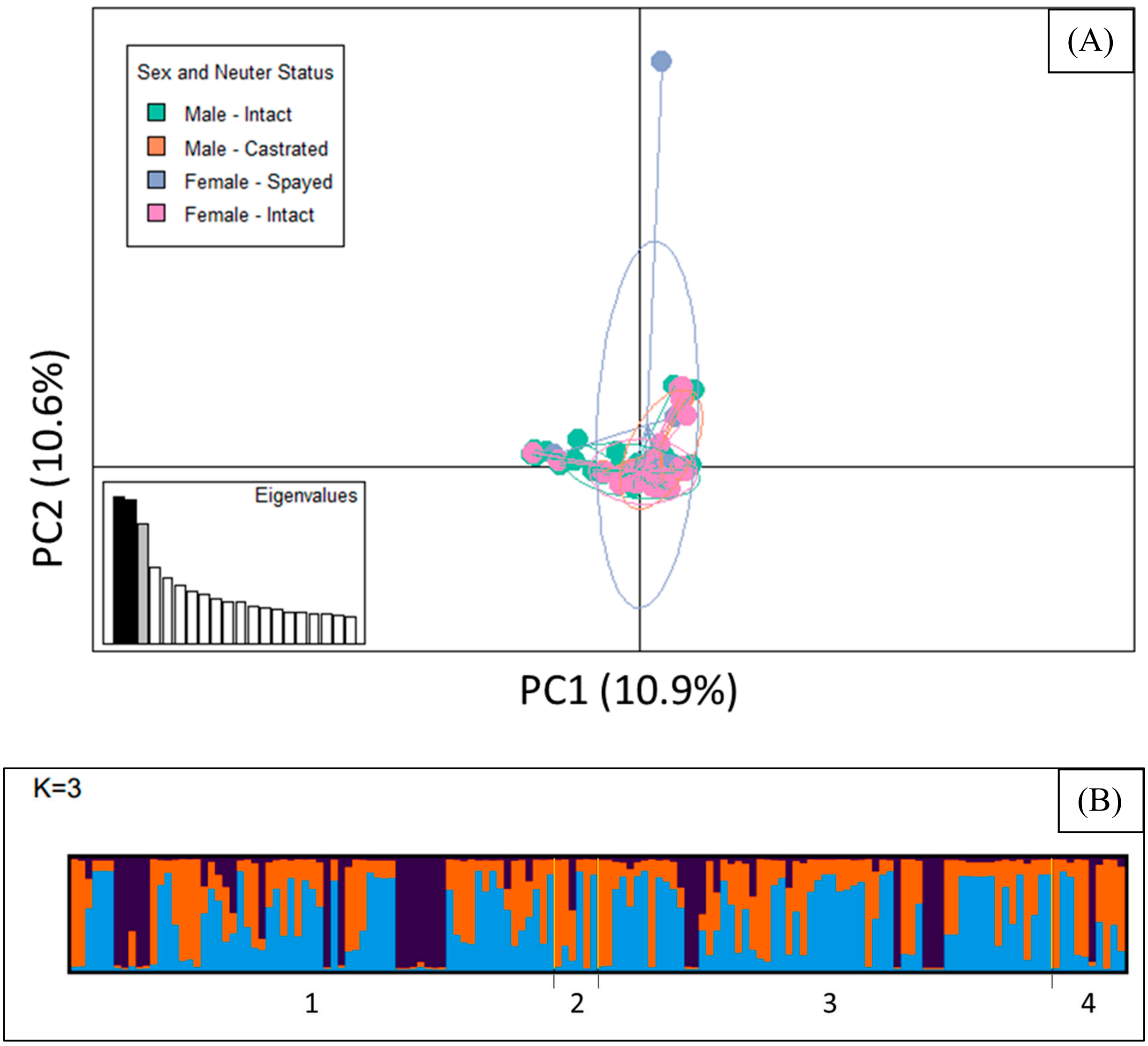

Figure S6. Genetic clustering is not observed among categories of sex and neuter status. (A) PCA plot for microsatellite regions using sex and neuter status collected from the clinical survey. The outlier point for female spayed group was a sample from MA. (B) STRUCTURE plot (k=3) grouping samples based on neuter status and sex where 1 = male intact, 2 = male castrated, 3 = female intact, 4 = female spayed. Locations included in study were California; New Mexico; Boulder County, CO; Larimer County, CO; and Massachusetts.
