# Supplementary material for: Genetic Characterization of Microsporum canis Clinical Isolates in the United States
Source: J Fungi (Basel). 2022 Jun 28;8(7):676. doi: 10.3390/jof8070676 (PMC9321804; doi:10.3390/jof8070676)
Supplement: Supplementary file 1 [file jof-08-00676-s001.zip › Supplementary Tables Moskaluk 2022.pdf]

Table S1. Survey provided to shelters and clinics for suspected dermatophytosis patients.

#	Questions	Answer options
1	Please enter the name of the person collecting the sample	Free response
2	Please select your clinic	Free response
3	Patient ID and name	Free response
4	Cat research ID	Free response
5	Date presented for ringworm evaluation	Free response
6	Cat's age	• Kitten – less than 1 year old<br>• Adult – over 1 year old
7	Cat's sex and neuter status	• Female – spayed<br>• Female – intact<br>• Female – unknown<br>• Male – castrated<br>• Male intact
8	Cat's breed	Free response
9	Cat's previous housing environment	• Stray<br>• Single cat – owned household<br>• Multi cat – owned household<br>• Previously owned – other animals unknown<br>• Transferred from shelter
10	Cat's current housing environment	• Shelter/rescue – single cat kennel<br>• Shelter/rescue – multiple cat kennel<br>• Client owned – single cat household<br>• Client owned – multiple cat household
11	Is this cat currently receiving any medications (excluding vaccines)?	• Yes – topical parasite preventatives or dewormer<br>• Yes – oral parasite preventatives or dewormer<br>• Yes – other<br>• No
12	Any other concurrent medical conditions?	Free response
13a	Have diagnostics for ringworm been performed? (select all that apply)	• Wood's lamp<br>• Fungal culture<br>• IDEXX PCR<br>• Hair observed under microscope<br>• Other<br>• None
13b	If diagnostics have been performed, what were the results?	Free response

14a	Has anti-fungal treatment been performed prior to sample collection?	• Yes<br>• No
14b	If yes, what treatment and for how long?	Free response
15	How many lesions are present?	• 1<br>• 2-5<br>• 5+<br>• None – no lesions present
16	What is the average size of the lesions?	• Less than 0.5 inch (smaller than a dime)<br>• Between 0.5 inch and 1 inch (size of a penny)<br>• Greater than 1 inch (larger than a penny)<br>• N/A – no lesions present
17	Lesion distribution (select all that apply)	• Head<br>• Neck<br>• Abdomen<br>• Back<br>• Tail including base of tail<br>• Legs including paws<br>• N/A – no lesions present
18	Lesion types (select all that apply)	• Alopecia<br>• Scales/crusts<br>• Reddened/erythematous<br>• Papules/pustules<br>• Miliary dermatitis<br>• N/A – no lesions present
19	Rate the severity of the lesions	• Mild – lesions are small and confined to focal area<br>• Mild to moderate<br>• Moderate – lesions are found in multiple areas, but is not widespread<br>• Moderate to severe<br>• Severe – lesions are large and found all over the body<br>• N/A – no lesions present
20	Please upload pictures of lesions/alopecia, Wood's lamp or trichogram	Free response
21	Any additional comments?	Free response

Questions with multiple choice answers have all possible answers shown while questions that could have answers written in are listed as free response. N/A = not applicable.

Table S2. Primers utilized for characterizing SUB3 and SSU1 genes.

Target	Pair	Orientation	Primer sequence 5' to 3'	Reference
SSU1	1	Forward	CAACCCAAGGATCCCAGGTC	This paper
		Reverse	CGGGTGATCGAGATGGTGAG	
SSU1	2	Forward	GAGCTTCAGGTCACAGCCAT	This paper
		Reverse	GCATAGAATGGGCGGGATGA	
SSU1	3	Forward	CTCACCATCTCGATCACCCG	This paper
		Reverse	CGGATCCCCACATCACGTAG	
SSU1	4	Forward	TACCTCTTCGCTCGAGACCA	This paper
		Reverse	ACTCCTTGCCCATCTGTGTG	
SSU1	5	Forward	CCCATTGCCGGTGAGATCTT	This paper
		Reverse	GCGTTTATCGTCGAAGGGGA	
SSU1	6	Forward	CACACAGATGGGCAAGGAGT	This paper
		Reverse	CGGGGTTTTCCTCTGAAGCT	
SUB3	1	Forward	GTTCGAAAAGACGCGCTTTCTTTCACAACCGT	[Descamps *et al.* 2002]
		Reverse	GTAGCCCTATCTTCCACTTCCGTTGTA	

Table S3. Clinical presentation data for *Microsporum canis* positive cats from CA, NM, CO (Boulder, Larimer), and MA.

Clinical Parameters	CA (n=109)	NM (n=31)	BC (n=6)	LC (n=4)	MA (n=4)
Age	Kitten (99 %)	Kitten (97 %)	Kitten (100 %)	Kitten (100 %)	Kitten (25 %)
Sex	Female (49 %)	Female (45 %)	Female (50 %)	Female (50 %)	Female (50 %)
Neuter status	Intact (91 %)	Intact (94 %)	Intact (66.7 %)	Intact (100 %)	Intact (25 %)
Breed	DSH (92.6 %)<br>DMH (4.6 %)<br>DLH (2.8 %)	DSH (84 %)<br>DMH (16 %)	DSH (83.3 %)<br>DMH (16.6 %)	DSH (100 %)	DSH (25 %)<br>DLH (25 %)<br>Persian (25 %)<br>Exotic shorthair (25 %)
Previous housing environment<br>1. Stray<br>2. Single cat – owned household<br>3. Multi cat – owned household<br>4. Previously owned – other animals unknown<br>5. Transferred from shelter	1 (98 %)<br>4 (1 %)<br>5 (1 %)	1 (100 %)	4 (16.6 %)<br>5 (83.3 %)	5 (100 %)	3 (75 %)<br>5 (25 %)
Current housing environment<br>1. Shelter/rescue – single cat kennel<br>2. Shelter/rescue – multiple cat kennel<br>3. Client owned – single cat household<br>4. Client owned – multiple cay household	1 (30 %)<br>2 (69 %)<br>3 (1 %)	1 (19 %)<br>2 (81 %)	1 (16.6 %)<br>2 (83.3 %)	2 (100 %)	4 (100 %)
Current medications*<br>1. Yes – topical parasite preventatives or dewormer	1 (27.5 %)<br>2 (18.3 %)<br>3 (2 %)<br>4 (71.5 %)	2 (100 %)	2 (100 %)	4 (100 %)	1 (50 %)<br>3 (25 %)<br>4 (25 %)

2. Yes – oral parasite preventatives or dewormer<br>3. Yes – other<br>4. No					
Concurrent medical conditions*	URI (24 %)<br>ectoparasites (13 %)<br>Diarrhea (1 %)<br>Otitis media (1 %)<br>Degloved chin (1 %)<br>Underweight (1 %)<br>None (67 %)	URI (6.5 %)<br>Ectoparasites (3.2 %)<br>None (90 %)	None (100 %)	None (100 %)	IBD (25 %)<br>superficial pyoderma (25 %)<br>None (75 %)
Diagnostics performed*<br>1. Wood's lamp<br>2. Fungal culture<br>3. IDEXX PCR<br>4. Hair observed under microscope<br>5. Other<br>6. None	1 (99 %)<br>6 (1 %)	1 (93.5 %)<br>5 (6.5 %)	1 (100 %)<br>2 (100 %)	1 (100 %)<br>2 (100 %)	1 (100 %)<br>3 (100 %)<br>4 (50 %)<br>5 (25 %)
If diagnostics have been performed, what were the results?	Wood's lamp positive (88 %)	Wood's lamp positive (92.9 %)	Wood's lamp positive (50 %)	Wood's lamp positive (100 %)	Wood's lamp positive (75 %)
Number of lesions<br>1. 1<br>2. 2-5<br>3. 5+<br>4. None – no lesions present	1 (16.5 %)<br>2 (42.2 %)<br>3 (36.7 %)<br>4 (4.6 %)	1 (29 %)<br>2 (51.6 %)<br>3 (6.5 %)<br>4 (12.9 %)	1 (16.6 %)<br>2 (33.3 %)<br>4 (50 %)	2 (75 %)<br>3 (25 %)	1 (25 %)<br>3 (75 %)
Size of lesions<br>1. Less than 0.5 inch (smaller than a dime)<br>2. Between 0.5 inch and 1 inch (size of a penny)	1 (36.7 %)<br>2 (44 %)<br>3 (14.7 %)<br>4 (4.6 %)	1 (64.5 %)<br>2 (22.6 %)<br>4 (12.9 %)	1 (16.6 %)<br>2 (16.6 %)<br>3 (16.6 %)<br>4 (50 %)	2 (50 %)<br>3 (50 %)	1 (25 %)<br>2 (25 %)<br>3 (50 %)

3. Greater than 1 inch (larger than a penny)<br>4. N/A – no lesions present					
Lesion distribution*<br>1. Head<br>2. Neck<br>3. Abdomen<br>4. Back<br>5. Tail including base of tail<br>6. Legs including paws<br>7. N/A – no lesions present	1 (88 %)<br>2 (33 %)<br>3 (29 %)<br>4 (24 %)<br>5 (25 %)<br>6 (58 %)<br>7 (4.6 %)	1 (64.5 %)<br>2 (12.9 %)<br>3 (29 %)<br>4 (6.5 %)<br>5 (3.2 %)<br>6 (32.2 %)<br>7 (12.9 %)	1 (16.6 %)<br>2 (16.6 %)<br>3 (33.3 %)<br>7 (50 %)	1 (100 %)<br>2 (50 %)<br>4 (25 %)<br>6 (100 %)	1 (75 %)<br>2 (75 %)<br>3 (75 %)<br>4 (50 %)
Lesion types*<br>1. Alopecia<br>2. Scales/crusts<br>3. Reddened/erythematous<br>4. Papules/pustules<br>5. Miliary dermatitis<br>6. N/A – no lesions present	1 (94.5 %)<br>2 (64 %)<br>3 (2.8 %)<br>5 (1.8 %)<br>6 (4.6 %)	1 (74 %)<br>2 (55 %)<br>3 (9.7 %)<br>6(12.9 %)	1 (50 %)<br>3 (33.3 %)<br>6 (50 %)	1 (100 %)<br>2 (100 %)	1 (100 %)<br>2 (100 %)<br>3 (25 %)
Disease severity<br>1. Mild – lesions are small and confined to focal area<br>2. Mild to moderate<br>3. Moderate – lesions are found in multiple areas, but is not widespread<br>4. Moderate to severe<br>5. Severe – lesions are large and found all over the body<br>6. N/A – no lesions present	1 (24.8 %)<br>2 (13.8 %)<br>3 (31.2 %)<br>4 (7.3 %)<br>5 (18.3 %)<br>6 (4.6 %)	1 (61.3 %)<br>2 (9.7 %)<br>3 (12.9 %)<br>4 (3.2 %)<br>6 (12.9 %)	1 (33.3 %)<br>2 (16.6 %)<br>6 (50 %)	4 (100 %)	1 (25 %)<br>2 (25 %)<br>3 (25 %)<br>4 (25 %)

No clinical data was collected for Weld County, CO. CA = California, NM = New Mexico, BC = Boulder County CO, LC = Larimer County CO, MA = Massachusetts, DSH = domestic shorthair, DMH = domestic medium hair, DLH = domestic long hair, N/A = not applicable, URI = upper respiratory infection, IBD = inflammatory bowel disease. * = Percentages are greater than 100 as patients can have more than one answer.

Table S4. Allele sizes for microsatellite regions one to eight (MS1, MS2, MS3, MS4, MS5, MS6, MS7, MS8) for *Microsporum canis* samples.

Sample ID	MS1 (bp)	MS2 (bp)	MS3 (bp)	MS4 (bp)	MS5 (bp)	MS6 (bp)	MS7 (bp)	MS8 (bp)	Genotype	Mating gene type
BC1	117	97	116	157	102	109	127	116	DF	MAT1-1
BC2	117	97	116	155	102	109	127	116	DD	MAT1-1
BC3	117	97	116	159	102	111	125	116	DI	MAT1-1
BC4	117	97	116	155	106	109	127	116	DE	MAT1-1
BC5	117	97	116	155	102	109	127	116	DD	MAT1-1
BC6	117	97	116	155	100	109	127	116	DC	MAT1-1
CA1	113	97	114	155	102	109	125	116	O	MAT1-1
CA2	115	97	114	155	106	109	125	116	BC	MAT1-1
CA3	115	99	114	155	102	109	125	116	CS	MAT1-1
CA4	115	99	114	157	102	109	125	116	CV	MAT1-1
CA5	115	97	114	155	100	109	125	116	AT	MAT1-1
CA6	115	97	114	155	106	109	125	116	BC	MAT1-1
CA7	115	97	114	155	100	109	125	116	AT	MAT1-1
CA8	115	97	114	155	100	109	125	116	AT	MAT1-1
CA9	115	97	114	157	100	109	125	116	BK	MAT1-1
CA10	115	97	114	155	100	109	125	116	AT	MAT1-1
CA11	NA	101	NA	155	106	109	125	112	DS	MAT1-1
CA12	105	101	NA	155	96	109	125	112	F	MAT1-1
CA13	NA	101	NA	155	104	105	125	112	DR	MAT1-1
CA14	105	99	NA	155	106	109	125	112	E	MAT1-1
CA15	NA	101	NA	153	106	109	125	112	DQ	MAT1-1
CA16	115	97	114	161	102	113	123	116	CI	MAT1-1
CA17	115	97	114	155	100	107	125	116	AS	MAT1-1
CA18	115	97	114	155	106	109	125	116	BC	MAT1-1
CA19	115	97	114	159	100	109	123	116	BY	MAT1-1
CA20	115	97	114	NA	100	107	123	116	CK	MAT1-1
CA21	115	97	114	159	106	109	123	116	CC	MAT1-1
CA22	115	97	114	155	102	109	125	116	AW	MAT1-1
CA23	113	99	NA	155	98	109	125	112	U	MAT1-1
CA24	117	97	116	139	102	109	125	116	DA	MAT1-1
CA25	113	97	114	157	102	105	125	116	R	MAT1-1
CA26	115	97	114	159	102	107	123	116	CA	MAT1-1
CA27	115	97	114	161	102	107	123	NA	CF	MAT1-1
CA28	113	95	114	159	102	107	123	116	J	MAT1-1
CA29	115	97	114	155	102	NA	125	116	AZ	MAT1-1
CA30	115	97	114	155	98	NA	125	116	AR	MAT1-1
CA31	113	97	114	153	102	109	125	116	M	MAT1-1

CA32	115	97	114	NA	100	109	NA	116	CL	MAT1-1
CA33	113	97	114	155	102	107	125	116	N	MAT1-1
CA34	113	99	114	155	102	109	125	116	T	MAT1-1
CA35	113	97	114	155	102	109	125	116	O	MAT1-1
CA36	113	97	114	155	102	109	125	116	O	MAT1-1
CA37	115	97	114	155	98	109	125	116	AN	MAT1-1
CA38	NA	99	NA	153	96	109	125	112	DJ	MAT1-1
CA39	NA	99	NA	153	98	109	125	112	DK	MAT1-1
CA40	115	97	114	157	98	109	125	112	BE	MAT1-1
CA41	115	97	114	157	98	111	125	112	BG	MAT1-1
CA42	115	97	NA	157	104	NA	NA	112	CO	MAT1-1
CA43	115	97	114	157	98	109	125	116	BF	MAT1-1
CA44	115	97	114	157	104	109	125	112	BT	MAT1-1
CA45	115	97	114	155	104	NA	123	116	BB	MAT1-1
CA46	115	97	116	155	106	NA	123	116	CN	MAT1-1
CA47	115	97	114	155	98	111	125	116	AO	MAT1-1
CA48	115	97	114	157	98	NA	125	112	BH	MAT1-1
CA49	115	97	114	159	100	NA	125	116	BZ	MAT1-1
CA50	115	97	114	157	102	NA	125	116	BR	MAT1-1
CA51	NA	99	NA	153	98	NA	125	112	DL	MAT1-1
CA52	NA	99	NA	153	98	109	125	112	DK	MAT1-1
CA53	115	97	114	153	100	109	125	116	AK	MAT1-1
CA54	115	97	114	153	102	113	125	116	AM	MAT1-1
CA55	115	97	114	153	102	109	125	116	AL	MAT1-1
CA56	115	97	114	103	100	107	125	116	W	MAT1-1
CA57	115	97	114	157	96	105	125	112	BD	MAT1-1
CA58	115	97	114	155	100	109	125	116	AT	MAT1-1
CA59	115	97	114	157	102	NA	125	116	BR	MAT1-1
CA60	115	97	114	159	98	NA	125	112	BW	MAT1-1
CA61	115	97	114	155	100	109	125	116	AT	MAT1-1
CA62	115	97	114	157	102	NA	125	116	BR	MAT1-1
CA63	115	97	114	103	100	NA	125	116	X	MAT1-1
CA64	115	97	114	157	100	109	125	116	BK	MAT1-1
CA65	115	97	114	157	100	109	123	116	BI	MAT1-1
CA66	115	97	114	161	102	107	123	116	CE	MAT1-1
CA67	115	97	114	159	100	107	121	NA	BX	MAT1-1
CA68	115	97	114	155	100	107	125	116	AS	MAT1-1
CA69	115	99	114	103	102	107	125	116	CP	MAT1-1
CA70	115	97	114	155	98	NA	125	112	AQ	MAT1-1
CA71	115	97	114	155	102	111	125	116	AX	MAT1-1
CA72	115	97	114	155	102	NA	125	116	AZ	MAT1-1

CA73	115	97	114	155	102	107	125	116	AV	MAT1-1
CA74	115	97	114	155	98	NA	121	NA	AP	MAT1-1
CA75	115	97	114	103	102	107	125	116	Y	MAT1-1
CA76	115	97	114	155	102	105	125	116	AU	MAT1-1
CA77	115	97	114	155	102	NA	125	116	AZ	MAT1-1
CA78	115	97	114	155	102	NA	125	116	AZ	MAT1-1
CA79	115	97	114	157	102	NA	123	116	BQ	MAT1-1
CA80	111	99	114	157	102	107	125	116	I	MAT1-1
CA81	115	97	114	157	102	107	125	116	BM	MAT1-1
CA82	115	97	114	157	102	NA	125	116	BR	MAT1-1
CA83	115	97	114	157	102	NA	125	116	BR	MAT1-1
CA84	115	97	114	157	102	115	125	116	BP	MAT1-1
CA85	115	99	114	157	102	107	125	116	CU	MAT1-1
CA86	115	97	114	157	102	109	125	116	BO	MAT1-1
CA87	115	97	114	157	102	109	125	116	BO	MAT1-1
CA88	115	97	114	157	102	107	125	116	BN	MAT1-1
CA89	115	97	114	157	102	115	125	116	BP	MAT1-1
CA90	115	97	114	155	100	107	125	116	AS	MAT1-1
CA91	115	97	114	157	102	NA	125	NA	BS	MAT1-1
CA92	117	97	116	157	102	113	125	116	DH	MAT1-1
CA93	115	97	114	155	102	111	125	116	AX	MAT1-1
CA94	NA	99	NA	155	98	109	125	112	DM	MAT1-1
CA95	115	97	114	157	102	NA	125	116	BR	MAT1-1
CA96	115	99	114	155	100	107	125	116	CR	MAT1-1
CA97	NA	99	NA	155	106	109	125	112	DO	MAT1-1
CA98	115	97	114	157	102	109	125	116	BO	MAT1-1
CA99	115	97	114	155	102	115	125	116	AY	MAT1-1
CA100	115	97	114	155	100	109	125	116	AT	MAT1-1
CA101	NA	99	NA	155	98	109	125	112	DM	MAT1-1
CA102	115	97	114	161	102	109	123	116	CG	MAT1-1
CA103	115	97	114	161	100	NA	123	116	CD	MAT1-1
CA104	115	99	114	155	102	NA	123	116	CT	MAT1-1
CA105	113	97	114	NA	100	NA	125	116	S	MAT1-1
CA106	115	97	114	157	106	105	123	116	BU	MAT1-1
CA107	115	97	114	127	102	109	125	116	AF	MAT1-1
CA108	115	97	114	155	102	109	125	116	AW	MAT1-1
CA109	115	97	114	155	102	NA	125	116	AZ	MAT1-1
CA110	115	97	114	155	102	NA	125	116	AZ	MAT1-1
CA111	115	97	114	155	102	NA	125	116	AZ	MAT1-1
CA112	105	99	NA	155	98	111	125	112	C	MAT1-1
CA113	105	101	NA	155	98	111	125	112	G	MAT1-1

CA114	105	99	NA	155	98	NA	125	112	D	MAT1-1
CA115	NA	99	NA	155	98	NA	125	112	DN	MAT1-1
CA116	105	99	NA	155	98	107	125	112	B	MAT1-1
CA117	105	99	NA	155	98	NA	125	112	D	MAT1-1
CA118	NA	99	NA	155	98	109	125	112	DM	MAT1-1
CA119	105	99	NA	155	98	NA	125	112	D	MAT1-1
CA120	NA	101	NA	153	98	111	125	112	DP	MAT1-1
CA121	105	99	NA	155	98	NA	125	112	D	MAT1-1
CA122	115	97	114	157	102	115	125	116	BP	MAT1-1
LC1	115	97	114	119	100	111	125	116	AD	MAT1-1
LC2	115	97	114	119	100	111	125	116	AD	MAT1-1
LC3	115	97	114	119	100	111	125	116	AD	MAT1-1
LC4	113	97	114	119	106	111	125	116	L	MAT1-1
MA1	117	97	116	141	102	105	125	116	DB	MAT1-1
MA2	115	97	116	141	102	109	127	116	CM	MAT1-1
MA3	109	97	110	121	104	109	123	118	H	MAT1-1
MA4	115	97	114	157	100	111	123	116	BL	MAT1-1
MA5	113	97	114	157	102	105	125	116	R	MAT1-1
NJ1	115	97	114	121	102	109	123	116	AE	MAT1-1
NM1	115	97	114	157	106	109	125	116	BV	MAT1-1
NM2	115	97	114	155	106	109	125	116	BC	MAT1-1
NM3	115	97	114	155	100	109	125	116	AT	MAT1-1
NM4	115	97	114	155	106	109	125	116	BC	MAT1-1
NM5	115	97	114	137	106	109	125	116	AJ	MAT1-1
NM6	115	97	114	155	100	109	125	116	AT	MAT1-1
NM7	115	97	114	155	106	109	125	116	BC	MAT1-1
NM8	115	97	114	155	102	107	125	116	AV	MAT1-1
NM9	115	97	114	155	106	109	125	116	BC	MAT1-1
NM10	105	97	114	155	100	109	125	116	A	MAT1-1
NM11	115	97	114	161	106	111	125	116	CJ	MAT1-1
NM12	115	97	114	161	106	111	125	116	CJ	MAT1-1
NM13	115	97	114	161	102	109	125	116	CH	MAT1-1
NM14	115	101	114	155	102	109	125	116	CY	MAT1-1
NM15	115	97	114	137	100	109	125	116	AG	MAT1-1
NM16	115	97	114	137	104	109	125	116	AI	MAT1-1
NM17	115	97	114	137	102	109	125	116	AH	MAT1-1
NM18	115	97	114	157	102	109	125	116	BO	MAT1-1
NM19	115	101	114	159	102	107	125	116	CZ	MAT1-1
NM20	115	97	114	159	102	NA	125	116	CB	MAT1-1
NM21	115	97	114	157	100	109	125	114	BJ	MAT1-1
NM22	115	97	114	157	102	109	125	116	BO	MAT1-1

NM23	115	97	114	157	102	109	125	116	BO	MAT1-1
NM24	115	99	114	159	102	109	125	116	CW	MAT1-1
NM25	115	97	114	157	102	109	125	116	BO	MAT1-1
NM26	115	97	114	157	102	109	125	116	BO	MAT1-1
NM27	115	99	116	157	102	111	NA	116	CX	MAT1-1
NM28	115	97	114	157	102	109	125	116	BO	MAT1-1
NM29	115	97	114	155	102	109	125	116	AW	MAT1-1
NM30	115	97	114	155	98	109	125	116	AN	MAT1-1
NM31	115	99	114	155	102	109	125	116	CS	MAT1-1
NM32	113	97	114	105	104	NA	123	116	K	MAT1-1
NM33	113	97	114	157	100	NA	NA	116	Q	MAT1-1
NM34	115	95	114	155	100	111	123	116	V	MAT1-1
NM35	115	97	114	155	104	109	125	116	BA	MAT1-1
NM36	113	97	114	157	98	109	123	116	P	MAT1-1
WC1	117	97	116	157	102	111	125	116	DG	MAT1-1
WC2	115	97	114	105	102	109	125	116	AA	MAT1-1
WC3	115	97	114	105	102	109	125	116	AA	MAT1-1
WC4	115	97	114	105	102	107	125	116	Z	MAT1-1
WC5	115	99	114	103	102	109	125	116	CQ	MAT1-1
WC6	115	97	114	105	102	NA	125	116	AB	MAT1-1
CBS113480	115	97	114	107	104	109	117	116	AC	MAT1-1

Of the clinical samples, 122 unique genotypes were observed with genotypes AT and BO being the most common (n=9 for each genotype). Samples were from Boulder County, CO (BC); California (CA); Larimer County, CO (LC); Massachusetts (MA); New Jersey (NJ); New Mexico (NM); and Weld County, CO (WC). CBS113480 isolate was included as a reference strain. NA = missing genotype.

Table S5. Clinic location and disease severity were significantly associated with genetic distance.

Clinical parameter	Degrees of Freedom	Sum of Squares	F	P-value
Clinic Location	4	3.0954	4.3127	0.001*
Sex/Intact Status	3	0.6237	1.1586	0.253
Size of Lesions	3	0.7176	1.3332	0.141
Number of Lesions	1	0.1633	0.9099	0.463
Disease Severity	1	0.6206	3.4587	0.004*
Previous Housing	3	0.7955	1.4778	0.139

Analysis of variance of the distance-based redundancy analysis. * = P-values were considered significant if less than 0.05). Samples from California; Boulder County, CO; Larimer County, CO; Massachusetts; and New Mexico were included in this analysis.

Table S6. Clinic location and disease severity were significantly associated with genetic distance.

Clinical parameter	Degrees of Freedom	Sum of Squares	F	P-value
Clinic Location	1	0.5337	2.998	0.012*
Sex/Intact Status	3	0.7643	1.431	0.127
Size of Lesions	3	0.5680	1.0634	0.348
Number of Lesions	1	0.2162	1.2143	0.234
Disease Severity	1	0.6009	3.3753	0.011*
Previous Housing	1	0.0774	0.4348	0.751

Analysis of variance of the distance-based redundancy analysis. * = P-values were considered significant if less than 0.05). Samples from California and New Mexico were included in this analysis as they had more than 10 individuals per clinic.
